# Effectiveness of piperonyl butoxide and pyrethroid-treated long-lasting insecticidal nets (LLINs) versus pyrethroid-only LLINs with and without indoor residual spray against malaria infection: third year results of a cluster, randomised controlled, two-by-two factorial design trial in Tanzania

**DOI:** 10.1186/s12936-023-04727-8

**Published:** 2023-10-03

**Authors:** Natacha Protopopoff, Jacklin F. Mosha, Louisa A. Messenger, Eliud Lukole, Jacques D. Charlwood, Alexandra Wright, Enock Kessy, Alphaxard Manjurano, Franklin W. Mosha, Immo Kleinschmidt, Mark Rowland

**Affiliations:** 1 Department of Disease Control, London School of Hygiene and Tropical Medicine, London, UK; 2 Mwanza Medical Research Centre, National Institute for Medical Research, Mwanza, Tanzania; 3 Kilimanjaro Christian Medical University College, Moshi, Tanzania; 4 MRC International Statistics and Epidemiology Group, London School of Hygiene and Tropical Medicine, London, UK; 5 School of Pathology, Faculty of Health Sciences, University of Witwatersrand, Johannesburg, South Africa; 6 Southern African Development Community Malaria Elimination Eight Secretariat, Windhoek, Namibia

**Keywords:** Malaria, Prevalence, Randomized controlled trial, Piperonyl butoxide and pyrethroid-treated nets, Tanzania

## Abstract

**Background:**

After decades of success in reducing malaria through the scale-up of pyrethroid long-lasting insecticidal nets (LLINs), the decline in the malaria burden has stalled, coinciding with the rapid spread of pyrethroid resistance. In a previously reported study, nets treated with a pyrethroid and a synergist, piperonyl butoxide (PBO), demonstrated superior efficacy compared to standard pyrethroid LLINs (std-LLINs) against malaria. Evidence was used to support the public health recommendation of PBO-Pyrethroid-LLIN by the World Health Organization in 2018. This study looks at the third year of rollout of these nets in Muleba district, Tanzania to inform whether policy guidelines need to be updated.

**Methods:**

A four-group cluster randomized trial (CRT) using a two-by-two factorial design was carried out between January 2014 and December 2017. A total of 48 clusters, were randomized in a 1:1:1:1 ratio to the following treatment groups, each intervention being provided once in 2015: 1/std-LLIN; 2/PBO-pyrethroid LLIN; 3/std-LLIN + Indoor Residual Spraying (IRS) and 4/PBO-Pyrethroid-LLIN + IRS. During the third year follow-up, malaria infection prevalence in 80 children per cluster, aged 6 months to 14 years, was measured at 28- and 33-months post-intervention and analysed as intention-to-treat (ITT) and per protocol (PP). Mosquito collections were performed monthly in all clusters, using CDC light traps in 7 randomly selected houses per cluster.

**Results:**

At 28 and 33 months, study net usage among household participants was only 47% and 31%, respectively. In ITT analysis, after 28 months malaria infection prevalence among 7471 children was 80.9% in the two std-LLIN groups compared to 69.3% in the two PBO-Pyrethroid-LLIN (Odds Ratio: 0.45, 95% Confidence Interval: 0.21–0.95, p-value: 0.0364). After 33 months the effect was weaker in the ITT analysis (prevalence 59.6% versus 49.9%, OR: 0.60, 95%CI:0.32–1.13, p-value: 0.1131) but still evident in the PP analysis (57.2% versus 44.2%, OR: 0.34, 95%CI: 0.16–0.71, p-value: 0.0051). Mean number of *Anopheles* per night collected per house was similar between PBO-Pyrethroid-LLIN groups (5.48) and std-LLIN groups (5.24) during the third year.

**Conclusions:**

Despite low usage of PBO- Pyrethroid LLIN, a small impact of those nets on malaria infection prevalence was still observed in the 3rd year with the most protection offered to children still using them. To maximize impact, it is essential that net re-distribution cycles are aligned with this LLIN lifespan to maintain maximum coverage.

*Trial registration*: The trial was registered with ClinicalTrials.gov (registration number NCT02288637).

**Supplementary Information:**

The online version contains supplementary material available at 10.1186/s12936-023-04727-8.

## Background

Globally, significant progress has been made controlling malaria through the scale-up of pyrethroid long-lasting insecticidal nets (LLINs), indoor residual spraying (IRS) and other prevention, diagnostic and treatment tools. The World Health Organization (WHO) estimated that due to the implementation of large-scale interventions, 1.5 billion malaria cases and 7.6 million malaria deaths were averted, 68% of which being attributed to the use of insecticide-treated nets (ITNs) [1]. In recent years the decline in malaria cases has stagnated and in many countries, cases have increased due to various factors [2]. Insecticide resistance, particularly to pyrethroid insecticides, has become widespread, undermining the effectiveness of standard LLINs to control malaria [1]. In response, substantial investments have been made in the development of insecticides with novel modes of action, in an effort to improve malaria vector control and to potentially mitigate further selection for insecticide resistance [3].

Until 2017, pyrethroids were the only chemical recommended by the WHO for use in LLINs. The first new class of dual-active-ingredient LLINs were treated with a mixture of a pyrethroid insecticide and a synergist, piperonyl butoxide (PBO), which enhances pyrethroid toxicity by inhibiting the activity of metabolic enzymes, commonly over-expressed in resistant vector populations. PBO-Pyrethroid-LLINs received a WHO recommendation following evidence from the present study on the efficacy of those nets against malaria infection over 2 years of use [4]. In the present trial, the odds of malaria infection prevalence was reduced by 33% after 21 months of community use in PBO-Pyrethroid-LLINs compared to standard pyrethroid-only LLINs in areas of high pyrethroid resistance [4]. A second trial in Uganda, confirmed the results of our study and reported a reduction of 27% in malaria infection prevalence in PBO-Pyrethroid-LLINs users after 12 months [5], and 20% at 25 months [6]. A subsequent meta-analysis determined that PBO-Pyrethroid-LLINs reduced the odds of malaria infection by 31%, compared to standard pyrethroid-only LLINs 21–25 months after deployment [7]. A more recent community trial in Kenya demonstrated a 26% reduction in *Plasmodium falciparum* prevalence in children after one year of PBO-Pyrethroid-LLIN use [8]. PBO-Pyrethroid-LLINs are now being distributed by National Malaria Control Programmes (NMCPs) across sub-Saharan Africa, with over 96 million nets procured in 2021 [9]. A second generation of LLINs combining a pyrethroid and a pyrrole (chlorfenapyr) or insect growth regulator (pyriproxyfen) have also undergone cluster randomized trial (CRT) evaluations [10–12], with chlorfenapyr-pyrethroid LLIN reducing odds of malaria infection prevalence by 55% 2 years after LLIN distribution in Tanzania [13] and 40% after 18 months in Benin [14].

Net deployment regimens currently assume three years of functional survival for LLINs under field conditions [15]. However, multiple cohort studies of pyrethroid LLINs across Africa have demonstrated substantial heterogeneity in effective net lifespan, both within and between types of LLIN and endemic regions [16–18]. These observations reflect variations in hole accumulation and rates of insecticide decline, which while initially dictated by product textile and chemical features, are in turn exacerbated by differences in user behaviour and acceptability [17, 19]. Textile degradation can also directly affect net usage [19]. The deterioration characteristics of pyrethroid-only LLINs cannot be extrapolated to dual-active-ingredient LLINs, because partner synergists or insecticides may degrade faster than pyrethroids or interact in the netting to affect the diffusion of one or both active ingredients over time. A household randomized trial from Kenya reported more than 80% of PBO was lost from PBO-Pyrethroid-LLINs after 3 years of use compared to 50% of the pyrethroid insecticide [20].

Overall, there is a paucity of field data evaluating the effectiveness of PBO-Pyrethroid-LLINs to prevent malaria over longer periods of time. Their superiority and potential for cost-effectiveness over pyrethroid-only LLINs, while evident after 2 years in some endemic settings, may decline rapidly thereafter. In a recent CRT conducted in Misungwi a different district of Tanzania, the reduction in malaria infection prevalence between PBO-Pyrethroid-LLINs compared to standard LLINs was 45% after 1 year, while there was no further reduction observed in the second year [13]. There is clearly a need for large-scale longitudinal monitoring studies of PBO-Pyrethroid-LLINs in various settings. Crucial questions remain unanswered regarding the performance of PBO-Pyrethroid-LLINs after 24 months, which will have direct implications for the optimal frequency of their procurement, and their role in resistance management, in the current arsenal of dual-active-ingredient LLINs and IRS products. A secondary analysis of the present trial conducted in northern Tanzania investigated the textile durability of the PBO-Pyrethroid-LLINs and showed that median survival time for these nets in the study site was 1.6 years and that 97% of the PBO content was lost after 3 years [21] confirming findings of short life span of these nets from other studies [20, 22].

This paper reports on the effectiveness of PBO-Pyrethroid-LLINs compared to pyrethroid-only LLINs against malaria infection after 3 years of continuous community-use in an area of intense pyrethroid resistance.

## Methods

### Study design and participants

A four-group CRT was conducted using a two-by-two factorial design between January 2014 and December 2017 in 40 villages in Muleba district, Kagera region, northern Tanzania [4]. Details of the study and its main outcomes relating to two years of follow-up have been reported previously [4]. The main study concluded in 2016 but follow-up was extended for an additional year to assess the effectiveness of PBO-Pyrethroid-LLINs during a third year of use, corresponding to their expected full lifespan. During the first two years follow-up malaria infection prevalence in children under 15 years old varied between trial groups and survey timepoints, from 26 to 68%. High pyrethroid resistance intensity in all 3 local vector species *Anopheles gambiae *sensu stricto (*s.s*.), *Anopheles arabiensis* and *Anopheles funestus *sensu lato (*s.l*.) has been reported [23].

All households in the study area were eligible to receive the interventions. Only households situated in the cluster core area with children aged 6 months to 14 years were included in malaria and mosquito cross-sectional surveys, conducted in June and November every year between 2015 and 2017. Exclusion criteria included dwellings not found or vacant, unwillingness to give informed consent, or eligible children who were severely ill or who did not reside permanently in the household.

### Randomisation and masking

A total of 48 clusters, divided into an inner core area, and an outer buffer zone of 300 m minimum [24], were randomized in a 1:1:1:1 ratio to the following treatment groups: 1/standard LLIN; 2/PBO-Pyrethroid LLIN; 3/standard LLIN + IRS and 4/PBO-Pyrethroid LLIN + IRS (Additional file 1: Table S1). The randomization was done by an independent epidemiologist and balanced on the following three restriction variables recorded in the baseline survey: malaria infection prevalence in children aged 6 months to 14 years (maximum difference allowed between study groups ± 7%), mosquito net usage (± 10%) and households in the lowest socio-economic status tercile (± 10%). Of 200,000 random allocations 29,478 met the restriction criteria, and after verifying that clusters were independently allocated to study groups, one of the eligible allocations was randomly chosen [4].

The two LLINs were the same colour and shape and only distinguishable by label codes and different coloured thread stitch added during the manufacturing process. Participants and field staff collecting the data were blinded to the type of LLINs in each study group but not to the IRS treatment allocation.

### Intervention

Both IRS and LLIN distributions were conducted in February 2015. 45,000 standard LLIN, Olyset LLIN (Sumitomo Chemical, Japan), containing 20 g/kg of the pyrethroid permethrin, and 45,000 PBO-Pyrethroid-LLIN, Olyset Plus (Sumitomo Chemical, Japan), containing PBO (10 g/kg) and the pyrethroid permethrin (20 g/kg) were distributed in the allocated study groups to provide one LLIN per two persons. The organophosphate insecticide Actellic^®^ 300CS (Syngenta, Switzerland) containing microencapsulated pirimiphos-methyl, was sprayed onto the interior walls and ceiling or roof of each house at the recommended dosage of 1 g/m^2^, in the two IRS study groups. There were no further study interventions after February 2015 although study participants in all trial groups received standard pyrethroid LLIN (Olyset LLIN) top-ups through antenatal care clinics and school distributions, whenever they were eligible. Duration of efficacy of Actellic^®^ 300CS against wild mosquitoes, reported in experimental hut trials done in several countries, ranged between 3 to 9 months [25]. Annual IRS would have been necessary for a ‘valid comparison’ against LLINs whose WHO recommended lifespan is 3 years. In the present trial, by the third year the IRS sprayed in year one would be expected to have little or no residual efficacy. Permethrin and PBO concentration was assessed in a subset of LLINs, collected after 36 months of use in the community [21].

### Data collection

During each cross-sectional survey 55 households meeting the eligibility requirement were randomly sampled in each cluster. Household characteristics and information about number of residents, household wealth and mosquito net ownership and usage was collected using an electronic form programmed to run on personal digital assistants (PDAs) with Pendragon software (Universal version, Pendragon Software Corporation, Chicago, United States). In each household, up to three eligible children were selected at random for malaria *Plasmodium falciparum* infection testing using rapid diagnostic tests (CareStart™ (pf/PAN) Combo Test, DiaSys, Workingham, UK). Children found positive were treated with artemether–lumefantrine according to national guidelines. Haemoglobin concentration was also measured (HemoCue(R) Hb 201 + Ängelholm, Sweden) to assess anaemia. Children were checked for other symptoms and treated accordingly or referred to health facilities. The first (2015) and second year (2016) follow-up surveys have been previously reported [4]. This paper reports data from surveys conducted at 28 months (June 2017) and 33 months (November 2017) post LLIN distribution.

Monthly entomological cross-sectional surveys were conducted during the trial, with collections carried out between 3 January and 15 December 2017 for the third year. For each round, seven households per cluster were randomly selected and a Centers for Disease Control and Prevention (CDC) light trap was installed at the foot of a bed in one of the bedrooms and monitored for one night to assess vector density. Existing nets were not removed, but a new standard LLIN was hung up if the bed selected for CDC light trap had no net. Anophelines were morphologically identified to species or species complex and a PCR Taq Man assay was used to distinguish the two sibling species *An. gambiae s.s.* and *An. arabiensis* [26]. A subset of malaria vectors was tested for *P*. *falciparum* circumsporozoite protein (CSP) to estimate sporozoite prevalence and calculate entomological inoculation rate (EIR) [27].

### Outcomes

The primary outcome was malaria infection prevalence in children 6 month to 14 years of age measured in cross-sectional household surveys at 28- and 33-months post LLIN distribution. A secondary clinical outcome was the prevalence of severe anaemia (defined as haemoglobin < 8 g/dl) in children aged 6 months to 4 years old at the same time points, measured in the same cross-sectional surveys. The main entomological outcome was EIR, during the third year of follow-up, and other endpoints included vector population density and sporozoite prevalence assessed during the same period.

### Statistical analysis

A total of 48 clusters of 80 individuals per cluster provided 80% power to detect a 28% relative reduction in malaria infection prevalence between the combined IRS treatment groups (standard LLIN + IRS and PBO-Pyrethroid-LLIN + IRS groups, 24 clusters in total) and the two groups not receiving IRS (standard LLIN & PBO-Pyrethroid-LLIN, 24 clusters in total), and between the two PBO-Pyrethroid-LLIN groups (24 clusters of which 12 received IRS) compared to the two standard LLIN groups (24 clusters of which 12 received IRS), assuming a mean infection prevalence of 20% in the reference groups, and a coefficient of variation of 0.3 [4]. This sample size also allowed us to detect a 40% difference between any of the four individual study groups (12 clusters per group). Number of households for entomological monitoring was selected based on logistical constraints.

Differences in malaria infection prevalence in children under 15 years old and anaemia prevalence in children from 6 months to 4 years old between IRS groups versus no IRS groups, and PBO-Pyrethroid-LLIN groups versus standard pyrethroid-LLIN groups was conducted in an intention to treat (ITT) basis at each time point (28 and 33 months), using logistic regression allowing for within cluster correlation of responses by using a robust variance estimator to calculate standard errors. Interaction between IRS and PBO-Pyrethroid-LLINs was assessed during each survey. As study net usage was low, differences in malaria infection prevalence and anaemia prevalence were also analysed using a per-protocol (PP) analysis, restricted to only children using the allocated LLINs, for both outcomes. For the entomological outcomes, vector density and EIR, negative binomial regression with a robust variance estimator was used to estimate density rate ratios between groups after adjusting for baseline. EIR was estimated as the mean number of sporozoite infected *Anopheles* per house per night and weighted to account for the proportion of collected *Anopheles* processed for sporozoites. Sporozoite rate was compared using logistic regression. Statistical analysis was conducted using Stata (version 15).

To assess reduction in malaria infection prevalence over the 3 years, PBO-Pyrethroid-LLIN groups (with and without prior IRS) and standard pyrethroid-LLIN groups (with and without IRS) were compared. A post hoc ITT analysis was done using a logistic regression with a robust variance estimator. The model included fixed effects for survey time, study group, IRS/no IRS and the interaction time by study group. Data from all 6 malaria infection prevalence surveys conducted in June and November between 2015 and 2017 were included in this analysis.

## Results

**Fig. 1:**
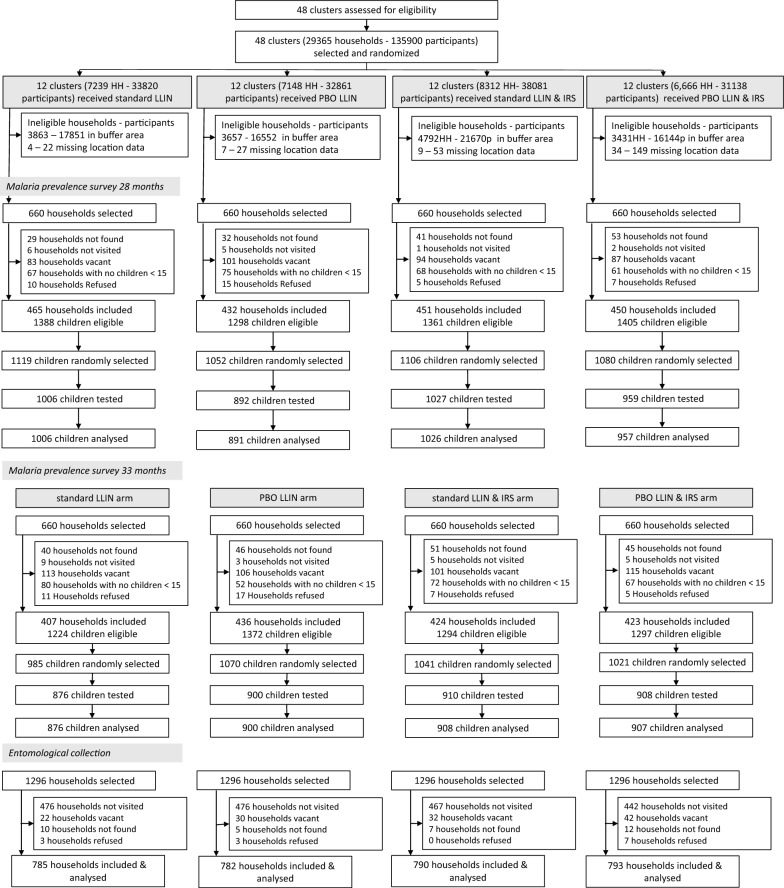
Trial profile

All 29,365 households received the allocated interventions (Fig. 1) in February 2015 and 13,568 were eligible to be enrolled for data collection; other households were in the buffer area or had missing information about their geolocations and were excluded. During each cross-sectional survey, 2640 households were selected, of which 68% (N = 1798) gave consent at t28 (14 June–11 July 2017) and 64% (N = 1680) at t33 (10 November–13 December 2017). The most common reasons not to participate was household members not being at home during the visit for 15% (N = 800) of responses, households with no children of eligible age (10%, N = 542) and households not found (6%, N = 337). Only 77 of the households refused to participate. A total of 4357 children were enrolled at 28 months, 89% (N = 3884) were tested for malaria infection prevalence, while the remaining children did not attend testing. At 33 months, 4117 were enrolled and 87% (N = 3594) were tested. Of the children tested in either of the cross-sectional surveys, 7471 were included in the analysis as 7 had missing malaria results. For mosquito collections, 5184 households were selected at random, of these 3150 (61%) gave consent; 1861 (36%) were not visited as the sample size of 8 households consented per cluster per round was met. The remaining households were either not found, nobody was home during the visit, or refused to participate (Fig. 1).

**Fig. 2:**
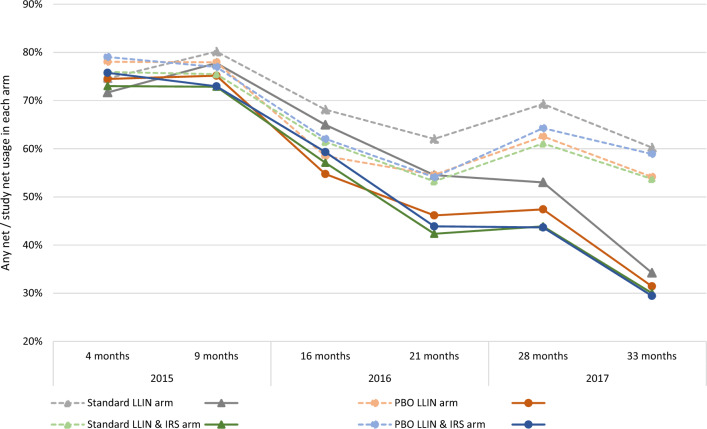
Net usage in all participants of any LLIN (interrupted line) and study LLIN (plain line) in each group over the study period

The baseline prevalence survey was conducted in September 2014. Results were presented previously [4], showing that children, household, and cluster characteristics were similar between trial groups (Table 1). Malaria infection prevalence in children aged 6 months to 14 years ranged from 61 to 68% between study arms which was within the limits set for the randomisation restriction criteria. Overall usage of study nets in the four groups dropped from 73% (3132/4312) 4 months post distribution to 47% (1847/3946) after 28 months, and further reduced to 31% (3031/9732) after 33 months with minor variation between study groups (highest usage: 34% for standard LLIN group vs lowest: 30% for the PBO-Pyrethroid-LLIN + IRS group) (Fig. 2; Additional file 1: Table S2). Study net usage in enrolled children was similar to that observed in other household members. Usage of any LLIN, which included study nets and standard LLINs received from routine distributions to children through primary school and to pregnant women during antenatal clinic visits, remained around 60% from 16 months onward. Permethrin remaining in nets after 36 months of use was 47% of the initial content in standard LLINs and 43% in PBO-Pyrethroid-LLINs, while only 4% of the PBO concentration was left (Additional file 1: Fig. S1) [21].

**Table 1:** Characteristics by study group at baseline

	Standard LLIN	PBO-Pyrethroid LLIN	Standard LLIN & IRS	PBO-Pyrethroid LLIN & IRS
Total population in core and buffer areas	33,820	32,861	38,081	31,138
Population in core area of clusters	15,947	16,282	16,358	14,845
Households in the lowest socioeconomic tercile	31.5% (27.2–35.7), 146/464	31.1% (27.1–35.0), 166/534	37.5% (33.3–41.6),198/528	34.9% (30.6–39.2), 163/467
Long-lasting insecticidal nets use in all participants	30.1% (28.5–31.7), 902/2996	26.3% (24.8–27.9), 810/3078	27.6% (26.1–29.2), 882/3197	26.3% (24.8–27.9), 810/3078
Malaria infection prevalence in children aged 6 months to 14 years, n/N	67.8% (64.7–70.9), 600/885	61.1% (58.1–64.2), 606/991	66.6% (64.0–69.5), 678/1018	63.6% (60.6–66.6), 615/967
Mean indoor vector per house per night, N	17.0 (0–34.7), 129	37.0 (4.0–70.1), 119	11.8 (0–24.7), 117	43.6 (9.7–77.6), 129

Data are expressed as mean or proportion % (95%CI) and the total sample size N except when stated otherwise

At 28 months post-intervention, malaria infection prevalence in the PBO-Pyrethroid-LLIN groups was lower compared to the standard LLIN groups (80.9%, versus 69.3%, Odds Ratio: 0.45, 95% Confidence Interval: 0.21–0.95, p: 0.0364) in the ITT analysis (Table 2). The effect was no longer significant at 33 months post distribution (58.6%, versus 49.9%, OR: 0.60, 95%CI (0.32–1.13), p: 0.1131). Similar findings were observed when comparing each of the PBO-Pyrethroid-LLIN groups individually to the standard LLIN group (Additional file 1: Table S3). Regarding children who slept under the allocated study nets in the PP analysis, those using PBO-Pyrethroid-LLINs had lower odds of malaria infection than those using a standard LLIN at both time points. There was also evidence of an interaction between PBO-Pyrethroid-LLINs and IRS at 33 months, suggesting that adding IRS to a background of standard LLIN may be more beneficial than adding it to a background of PBO-Pyrethroid-LLINs.

**Table 2:** Malaria infection prevalence in children 6 months to 14 years of age in the 3rd year follow up at 28 and 33 months post intervention

Intervention	Intention to treat					Per protocol^a^				
	n/N	%	OR	95%CI	P-value	n/N	%	OR	95%CI	P-value
**Survey 28 months**										
Standard LLIN^b^	1643/2032	80.9	1			762/962	79.2	1		
PBO-Pyrethroid LLIN^c^	1280/1848	69.3	0.45	0.21–0.95	0.0364	507/755	67.2	0.43	0.22–0.84	0.0151
No IRS^d^	1441/1897	76.0	1			663/881	75.3	1		
IRS^e^	1482/1983	74.7	0.79	0.44–1.41	0.4172	606/836	72.5	0.71	0.39–1.30	0.2615
Interaction coefficient			1.38	0.53–3.69	0.505			1.59	0.62–4.07	0.3239
**Survey 33 months**										
Standard LLIN^b^	1045/1784	58.6	1			291/509	57.2	1		
PBO-Pyrethroid LLIN^c^	901/1807	49.9	0.60	0.32–1.13	0.1131	200/453	44.2	0.34	0.16–0.71	0.0051
No IRS^d^	1009/1776	56.8	1			257/500	51.4	1		
IRS^e^	937/1815	51.6	0.52	0.25–1.07	0.0747	234/462	50.7	0.57	0.31–1.06	0.0736
Interaction coefficient			1.78	0.71–4.49	0.2131			3.11	1.18–8.20	0.0230

*OR* odds ratio for the factorial analysis compared the two-main intervention effect 1/ PBO-pyrethroid LLIN vs standard LLIN and 2/IRS vs no IRS and their interaction. OR unadjusted for baseline plasmodium infection prevalence

^a^Per protocol includes only children sleeping under the allocated nets

^b^Standard LLIN and standard LLIN & IRS groups

^c^PBO-Pyrethroid LLIN and PBO-Pyrethroid LLIN & IRS groups

^d^Standard LLIN and PBO-Pyrethroid LLIN groups

^e^standard LLIN & IRS and PBO-Pyrethroid LLIN & IRS groups

No difference was found in malaria infection prevalence between the IRS groups vs no IRS groups at any of the time points in the ITT analysis or the PP analysis (Table 2).

**Fig. 3:**
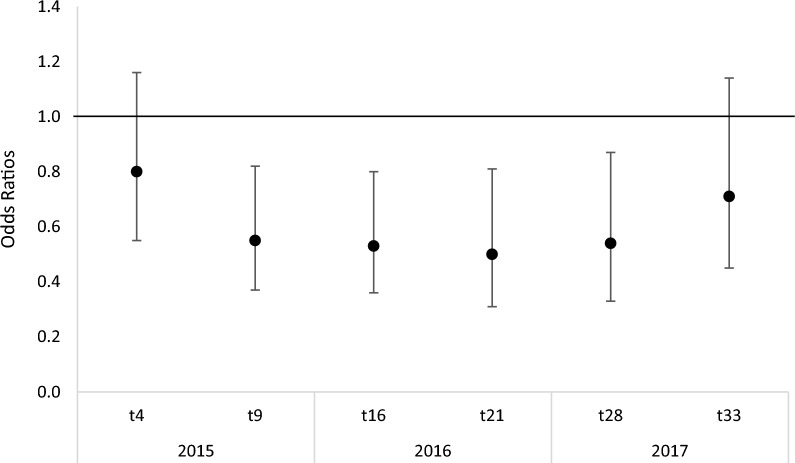
Effect trend of PBO-Pyrethroid LLIN groups (with and without IRS) on malaria prevalence compared to std LLINs groups (with and without IRS) during the 3 years follow up. An intention to treat analysis was done using a logistic regression with a robust variance estimator comparing PBO-Pyrethroid LLIN groups combined vs std LLIN groups combined and included fixed effects for time, study group, IRS and the interaction time by study group. P-value for interaction term was 0.0060

Similarly, no significant reduction in anaemia prevalence was found in children under 5 years old in any of the intervention groups (PBO-Pyrethroid-LLIN or IRS groups) vs their respective comparators (Additional file 1: Table S4). Over the 3 years follow up, malaria infection prevalence in the 3 intervention groups (LLIN + IRS, PBO-Pyrethroid-LLIN + IRS groups and, PBO-Pyrethroid-LLIN) was the lowest 9 months post intervention and increased afterward (Additional file 1: Fig. S2). However malaria infection prevalence in the two groups with PBO-Pyrethroid LLIN was consistently lower than standard LLIN groups (with and without IRS) with the stronger effect at 9, 16, 21, 28 months post intervention, while at 4 months and 33 months evidence was weaker (Fig. 3).

**Fig. 4:**
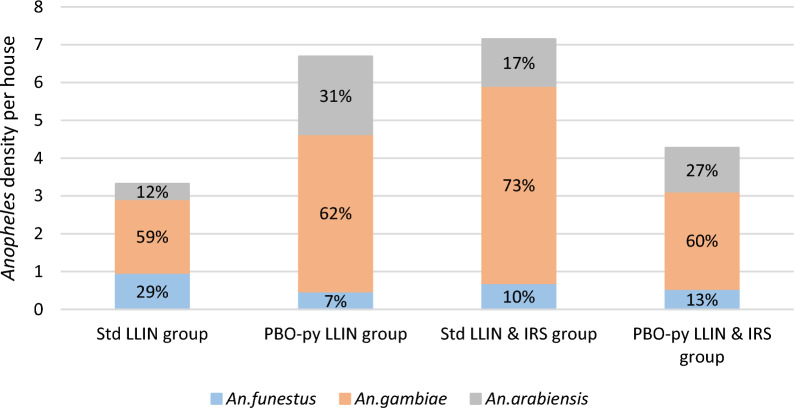
*Anopheles* densities per night per house for each vector species, proportion within group also presented in each of the bar chart

Through the 3rd year follow up, a total of 31,120 mosquitoes were collected during the 3150 collection nights, of which 56% (N = 17,451) were identified as *Anopheles.* Mean vector densities were 3.3 per house per night in the standard LLIN group, 6.7 in the PBO-Pyrethroid-LLIN group, 7.2 in the standard LLIN + IRS group and 4.3 in PBO-Pyrethroid-LLIN + IRS group (Fig. 4). The proportion of mosquitoes positive for *P. falciparum* sporozoites was 2.6% for the group PBO-Pyrethroid-LLIN + IRS and 4.9% and over for the other groups (Additional file 1: Table S5). In the factorial analysis there was no differences in vector density, sporozoite rate or EIR between PBO-Pyrethroid-LLIN vs standard LLIN groups or IRS vs no IRS groups during the third year follow-up (Table 3).

**Table 3:** Entomological outcomes comparing the two main interventions (PBO-Pyrethroid LLIN vs standard LLIN groups and IRS vs No IRS groups) in the 3rd year follow up in 2017

	Vector density per night per household					Sporozoite rate					EIR per nights per household				
	N	Mean	DR	95%CI	P-value	n/N	%	OR	95%CI	P-value	N	Mean^e^	DR^f^	95%CI	P-value
Standard LLIN^a^	1575	5.24	1			108/2070	5.2	1			1540	0.15	1		
PBO-Pyrethroid LLIN^b^	1575	5.48	1.59	0.80–3.16	0.1832	67/1788	3.8	0.88	0.55–1.42	0.5992	1536	0.11	0.63	0.25–1.61	0.3296
No IRS^c^	1567	5.00	1			103/1962	5.3	1			1524	0.14	1		
IRS^d^	1583	5.71	1.06	0.48–2.37	0.8807	72/1896	3.8	0.87	0.52–1.47	0.609	1552	0.12	0.97	0.41–2.28	0.9426
Interaction coefficient			0.47	0.16–1.40	0.1699			0.63	0.31–1.28	0.195			0.88	0.24–3.21	0.8489

Density ratio (DR) for vector density and EIR and odds ratio (OR) for sporozoite rate are adjusted for their respective baseline value

^a^Standard LLIN and standard LLIN & IRS groups

^b^PBO-Pyrethroid LLIN and PBO-Pyrethroid LLIN & IRS groups

^c^Standard LLIN and PBO-Pyrethroid LLIN groups

^d^Standard LLIN & IRS and PBO-Pyrethroid LLIN & IRS groups

^e,f^Arithmetic mean and DR of the EIR are weighted to account the proportion of mosquitoes sampled to be tested for sporozoites

No significant effect was observed when comparing each of the individual groups to standard LLINs (Additional file 1: Table S5). Overall 87.6% (14784/16884) of female vectors were *Anopheles gambiae s.l.* and the remaining (2100/16884) were *An. funestus s.l.*. Of the 892 *An. gambiae s.l.* tested by PCR to distinguish between sibling species, 75% were *An. gambiae s.s.* (N = 670) across the 4 groups whilst the highest proportion of *An. arabiensis* was found in the two groups with PBO-Pyrethroid-LLINs (Fig. 4).

## Discussion

In this CRT assessing the effectiveness of a new class of LLIN over 3 years, children residing in clusters that received PBO-Pyrethroid-LLINs had reduced odds of malaria infection compared to those who received standard LLINs up to 28 months post-intervention when half of the participants were still using the study nets. This effect became weaker and was no longer significant after a further 6 months when usage had dropped and most of the PBO content from the nets was lost. By the 36th month only 4% of the original PBO and half of the permethrin remained in the net [21]. However, the odds of infection in children still sleeping under the PBO-Pyrethroid-LLINs remained significantly less compared to those sleeping under standard LLIN, demonstrating the potential for PBO-Pyrethroid-LLINs to provide personal protection into their third year of community use at low concentrations of PBO. There was no reduction in vector density and malaria transmission (estimated through EIR) in the PBO-Pyrethroid-LLIN groups compared to standard LLIN groups. This contrasted with the first two years of the CRT where reductions were observed in both malaria infection prevalence and transmission in the PBO-Pyrethroid-LLIN groups [4], indicating a large community effect on vector population density and malaria prevention, when PBO-Pyrethroid-LLINs were less than 2 years old and usage was over 40%. Field studies have demonstrated the effect of high net coverage on mosquito population density and EIR [24], and modelling studies have showed that net usage as low as 35% can provide a mass effect [28]. In our study, as net usage dropped in the third year and concentrations of PBO and permethrin declined, the community effect might have been lost. Only individual protection remained, with the net providing a physical barrier and permethrin/PBO a repellence barrier against mosquito biting. This may explain the reduced impact of PBO-Pyrethroid-LLINs on malaria transmission in the community and the moderate protection observed mainly in children using PBO-Pyrethroid-LLINs. The loss of community protection was observed earlier in the Uganda trial [5, 6], where after 18 months, malaria reduction was seen only in PBO-Pyrethroid LLIN users.

Permethrin content in the PBO-Pyrethroid-LLINs and standard LLINs halved after 36 months, while PBO was reduced by 97% of the initial concentration [21]. This raises concerns about the long-term chemical durability of PBO and how long this new class of net will provide a superior effect compared to standard LLINs in areas of pyrethroid resistance.

In the first two years of our trial, an effect of PBO-Pyrethroid-LLINs on malaria transmission and vector density was observed when concentrations of PBO were reduced to 16% of the initial content. Personal protection against *Anopheles* bites was still observed in torn PBO-Pyrethroid-LLINs used for 21 months in the field [29], which may be due to higher excito-repellency of PBO-Pyrethroid-LLINs compared to standard LLINs [30]. Pyrethroid content remaining in nets was much lower in PBO-Pyrethroid-LLINs than standard Pyrethroid-LLINs after 24 months [21]. It has been suggested that higher pyrethroid release rates in this specific brand of PBO-Pyrethroid-LLIN (Olyset Plus) may increase the concentration of the insecticide on the surface of the net and hence the killing effect, regardless of PBO concentration [31]. The bioavailablity of insecticide on the net surface, instead of being trapped within the fibre, may explain the superior personal protection of Olyset Plus in the third year. The insecticidal contents were comparable to those reported after 36 months of use in a study conducted in Kenya [20] with the same brand of net. The faster reduction of PBO compared to pyrethroid was also observed in another community trial in Uganda [22] and in controlled studies on wash resistance of the net [31]. However, in the present trial, at the end of 36 months, permethrin contents were comparable between LLINs (9 g/kg and 10 g/kg in Olyset Plus vs Olyset LLIN, respectively). The superior effect of the PBO nets after 36 months was only seen in per protocol analysis, which unlike ITT analysis, cannot be assumed to be free of confounding. The analysis is limited by the selection bias and small number of children included in the PP analysis which was half of those in the ITT analysis at 28 months and only a quarter at 33 months. Alternatively, confounding may arise from factors unrelated to the nets, such as differences in health-seeking behaviour and socio-economic status of those still using the study nets at this time point.

The shorter than expected effective lifespan of PBO-Pyrethroid-LLINs and standard LLINs was also observed in a separate cohort of nets followed annually as part of this study showing that less than 17% of the nets distributed survived up to 3 years as assume. The development of holes in nets was the main driver of decreasing usage [21]. Other studies did not find a direct association between net textile deterioration and malaria [32], while level of net attrition was related to the accumulation of net wear and tear [19].

The WHO currently requires CRTs assessing the public health value new classes of LLINs to be conducted over a minimum of two transmissions seasons, while also expecting all classes of LLIN to demonstrate three years of working life. However, without good evidence relating to the longevity of LLINs procurement and distribution agencies are unlikely to change their assumption of a 3 year useful lifespan of nets to a more realistic replacement cycle of the product. NMCPs should therefore conduct routine monitoring and assessments of the impact of ageing of PBO-Pyrethroid-LLIN after their distributions as it will vary depending on brands and settings. The Tanzanian continuous delivery mechanism of LLINs through annual distributions to primary school children and antenatal consultations, rather than mass distribution campaigns, seems more in tune with the PBO-Pyrethroid-LLIN lifespan thereby maintaining good coverage [33] and a mixture of newer effective nets and aged net across the community. The development of a stronger net material would also prolong net usage as holes would develop more slowly. In addition, community sensitisation to improve care and repair could also reduce the rate of textile degradation [34], maintain higher coverage, and increase the required interval between distributions. Interventions that successfully improve these parameters will alter the relative cost-effectiveness of products and the viability of each strategy [35].

The absence of effect observed in the IRS group was expected as pirimiphos-methyl does not last on walls of houses for more than 9 months [25]. Yearly rounds of IRS are usually necessary to maintain efficacy. A modelling study showed that the effect of repeated IRS would have averted 45 more malaria cases per 1000 people per year over 3 years in the present trial study area, compared to PBO-Pyrethroid-LLINs alone, and this benefit would have been the highest in the third year, when net usage had decreased [35]. However, PBO-Pyrethroid-LLINs were still reported as being the most cost-effective intervention. Longer-lasting formulations of non-pyrethroid insecticides for IRS might be a more cost-effective alternative to existing IRS products. New IRS active ingredients (e.g. clothianidin and broflanilide) the have potential to be used in resistance management schemes with PBO-Pyrethroid-LLINs, if no antagonistic effects are present [36]. Deployment regimens for IRS and new dual-active-ingredient LLINs need to more accurately reflect the true effective lifespan of each product to avoid leaving both LLINs and IRS in communities past their optimum operational lifespans. Without timely replacement, vector populations may be exposed to sublethal insecticide doses with the potential for faster selection of insecticide resistance [37].

## Conclusions

PBO-Pyrethroid-LLINs may still provide some additional personal protection to users compared to those using standard LLINs in the third year when malaria infection prevalence remained high. However, this protection did not extend to non-users of PBO-Pyrethroid-LLINs through any mass effect. The declining efficacy and low usage of PBO-Pyrethroid LLINs after 2 years suggests that schedules replacing nets every three years are unlikely to control malaria adequately and will leave populations unprotected. For maximum impact, distribution regimens must be adapted to the lifespan of new classes of LLINs products coming into the market and/or the longevity of this product should be enhanced to meet the WHO recommended durability of three years.

### Supplementary Information

**Additional file 1: Table S1**: Factorial design. **Table S2**: Net coverage indicators during cross sectional surveys conducted in 2017, 28 and 33 months post intervention. **Figure S1**: Reduction in pyrethroid and PBO content over time. **Table S3**: Malaria infection prevalence in children 6 months to 14 years of aged at 28 and 33 months post intervention comparing each individual group to standard LLIN group as reference in intention to treat and per protocol analysis. **Table S4**: Anaemia prevalence (haemoglobin level < 8g/dl) in children under 5 years old at 28 and 33 months post intervention in intention to treat and per protocol analysis. **Figure S2**: Changes in prevalence overtime in each study group from 2014 (baseline survey pre-intervention) to 2017. **Table S5**: Effect of each individual interventions compared to standard LLIN on entomological outcomes.

## Data Availability

The datasets used and/or analysed during the current study are available from the corresponding author on reasonable request.

## Abbreviations

CDC: Centers for Disease Control
CRT: Cluster randomized trial
CSP: Circumsporozoite protein
EIR: Entomological inoculation rate
IRS: Indoor residual spraying
ITN: Insecticide-treated net
ITT: Intention-to-treat (ITT)
LLINs: Long lasting insecticidal net
NMCP: National Malaria Control Programme
PBO: Piperonyl butoxide
PP: Per protocol
s.l.: Sensu lato
s.s.: Sensu stricto
std-LLINs: Standard pyrethroid LLINs
