# Supplementary material for: Effectiveness of piperonyl butoxide and pyrethroid-treated long-lasting insecticidal nets (LLINs) versus pyrethroid-only LLINs with and without indoor residual spray against malaria infection: third year results of a cluster, randomised controlled, two-by-two factorial design trial in Tanzania
Source: Malar J. 2023 Oct 3;22:294. doi: 10.1186/s12936-023-04727-8 (PMC10548685; doi:10.1186/s12936-023-04727-8)
Supplement: Supplementary file 1 — Additional file 1: Table S1: Factorial design. Table S2: Net coverage indicators during cross sectional surveys conducted in 2017, 28 and 33 months post intervention. Figure S1: Reduction in pyrethroid and PBO content over time. Table S3: Malaria infection prevalence in children 6 months to 14 years of aged at 28 and 33 months post intervention comparing each individual group to standard LLIN group as reference in intention to treat and per protocol analysis. Table S4: Anaemia prevalence (haemoglobin level < 8g/dl) in children under 5 years old at 28 and 33 months post intervention in intention to treat and per protocol analysis. Figure S2: Changes in prevalence overtime in each study group from 2014 (baseline survey pre-intervention) to 2017. Table S5: Effect of each individual interventions compared to standard LLIN on entomological outcomes. [file 12936_2023_4727_MOESM1_ESM.docx]

**Additional file 1**

Effectiveness of piperonyl butoxide and pyrethroid-treated long-lasting insecticidal nets (LLINs) versus pyrethroid-only LLINs with and without indoor residual spray against malaria infection: third year results of a cluster, randomised controlled, two-by-two factorial design trial in Tanzania

Natacha Protopopoff^1*+^, Jacklin F. Mosha^2+^, Louisa A. Messenger^1^, Eliud Lukole^2^, Jacques D. Charlwood^1^, Alexandra Wright^1^, Enock Kessy^3^, Alphaxard Manjurano^2^, Franklin W. Mosha^3^, Immo Kleinschmidt^4,5^, Mark Rowland^1^

Table S1: Factorial design

|  | Standard pyrethroid LLIN | PBO-pyrethroid LLIN |
| --- | --- | --- |
| No IRS | group 1 = 12 clusters | group 2= 12 clusters |
| IRS | group 3 = 12 clusters | group 4 = 12 clusters |

Table S2: Net coverage indicators during cross sectional surveys conducted in 2017, 28 and 33 months post intervention.

* Any LN include study LLIN and other LLIN. Results are expressed in proportion with (95%CI) and absolute number n/N. N is total household number for IRS, LLIN ownership and LLIN access and total number of resident for usage.

Figure S1: Reduction in pyrethroid and PBO content over time

The figure shows the reduction in pyrethroid and PBO content compared to initial content (t0) in 10 nets of each type collected at 12 months (t12), 24 months (t24) and 36 months post interventions.

Table S3: Malaria infection prevalence in children 6 months to 14 years of aged at 28 and 33 months post intervention comparing each individual group to standard LLIN group as reference in intention to treat and per protocol analysis.

|  | Intention to treat | | | | | Per protocol* | | | | |
| --- | --- | --- | --- | --- | --- | --- | --- | --- | --- | --- |
| Intervention group | n/N | % | OR | 95%CI | P-value | n/N | % | OR | 95%CI | P-value |
| **Survey 28 months** |  |  |  |  |  |  |  |  |  |  |
| Standard LLIN group | 832/1006 | 82.7% | 1 |  |  | 427/522 | 81.8% | 1 |  |  |
| PBO-py LLIN group | 609/891 | 68.4% | 0.45 | 0.21-0.95 | 0.036 | 236/359 | 65.7% | 0.43 | 0.22-0.84 | 0.0151 |
| Standard LLIN & IRS group | 811/1026 | 79.0% | 0.79 | 0.44-1.41 | 0.417 | 335/440 | 76.1% | 0.71 | 0.39-1.30 | 0.2615 |
| PBO-py LLIN & IRS group | 671/957 | 70.1% | 0.49 | 0.25-0.97 | 0.040 | 271/396 | 68.4% | 0.48 | 0.25-0.94 | 0.0318 |
| **Survey 33 months** |  |  |  |  |  |  |  |  |  |  |
| Standard LLIN group | 568/876 | 64.8% | 1 |  |  | 169/265 | 63.8% | 1 |  |  |
| PBO-py LLIN group | 441/900 | 49.0% | 0.52 | 0.25-1.07 | 0.0747 | 88/235 | 37.5% | 0.34 | 0.16-0.71 | 0.0051 |
| Standard LLIN & IRS group | 477/908 | 52.5% | 0.60 | 0.32-1.13 | 0.1131 | 122/244 | 50.0% | 0.57 | 0.31-1.06 | 0.0736 |
| PBO-py LLIN & IRS group | 460/907 | 50.7% | 0.55 | 0.29-1.09 | 0.0851 | 112/218 | 51.4% | 0.60 | 0.30-1.22 | 0.1532 |

OR unadjusted for baseline plasmodium infection prevalence. * per protocol includes only children sleeping under the allocated nets.

Table S4: Anaemia prevalence (haemoglobin level <8g/dl) in children under 5 years old at 28 and 33 months post intervention in intention to treat and per protocol analysis

|  | Intention to treat | | | | | Per protocol* | | | | |
| --- | --- | --- | --- | --- | --- | --- | --- | --- | --- | --- |
| Intervention | n/N | % | OR | 95%CI | P-value | n/N | % | OR | 95%CI | P-value |
| **Survey 28 months** |  |  |  |  |  |  |  |  |  |  |
| Standard LLIN ^1^ | 66/556 | 11.9% | 1 |  |  | 37/307 | 12.1% | 1 |  |  |
| PBO-py LLIN ^2^ | 67/566 | 11.8% | 1.07 | 0.62-1.85 | 0.7968 | 38/287 | 13.2% | 1.27 | 0.63-2.56 | 0.4988 |
| No IRS ^3^ | 83/560 | 14.8% | 1 |  |  | 47/304 | 15.5% | 1 | 0.63-2.56 | 0.4988 |
| IRS ^4^ | 50/562 | 8.9% | 0.60 | 0.32-1.15 | 0.123 | 28/290 | 9.7% | 0.67 | 0.28-1.58 | 0.3468 |
| Interaction coefficient |  |  | 0.86 | 0.35-2.10 | 0.7395 |  |  | 0.76 | 0.23-2.51 | 0.6514 |
| **Survey 33 months** |  |  |  |  |  |  |  |  |  |  |
| Standard LLIN ^1^ | 30/489 | 6.1% | 1 |  |  | 10/168 | 6.0% | 1 |  |  |
| PBO-py LLIN ^2^ | 38/510 | 7.5% | 1.05 | 0.42-2.62 | 0.9203 | 12/146 | 8.2% | 1.37 | 0.39-4.81 | 0.6138 |
| No IRS ^3^ | 32/486 | 6.6% | 1 |  |  | 16/169 | 9.5% | 1 |  |  |
| IRS ^4^ | 36/513 | 7.0% | 0.90 | 0.41-1.99 | 0.799 | 6/145 | 4.1% | 0.43 | 0.12-1.50 | 0.181 |
| Interaction coefficient |  |  | 1.37 | 0.42-4.40 | 0.5944 |  |  | 0.96 | 0.15-6.18 | 0.9644 |

OR, odds ratio for the factorial analysis compared the two-main intervention effect 1/PBO LLIN vs No PBO LLIN and 2/IRS vs no IRS and their. OR unadjusted for baseline anaemia prevalence. * per protocol includes only children sleeping under the allocated nets.

^1^ Standard LLIN and standard LLIN & IRS groups, ^2^ PBO-pyrethroid LLIN and PBO-pyrethroid LLIN & IRS groups, ^3^ standard LLIN and PBO-pyrethroid LLIN groups, ^4^ standard LLIN & IRS and PBO-pyrethroid LLIN & IRS groups.

Fig. S2: Changes in prevalence overtime in each study group from 2014 (baseline survey pre-intervention) to 2017.

Table S5: Effect of each individual interventions compared to standard LLIN on entomological outcomes

|  | **Vector density per night per household** | | | | |  | | **Sporozoite rate** | | | | | |  | **EIR per night per household**** | | | | |
| --- | --- | --- | --- | --- | --- | --- | --- | --- | --- | --- | --- | --- | --- | --- | --- | --- | --- | --- | --- |
|  | N | Mean | DR* | 95%CI | P-value | |  | | n/N | % | OR* | 95%CI | P-value |  | N | Mean | DR* | 95%CI | P-value |
| Standard LLIN group | 785 | 3.32 | 1 |  |  | |  | | 60/1093 | 5.5% | 1 |  |  |  | 767 | 0.15 | 1 |  |  |
| PBO-py LLIN group | 782 | 6.69 | 1.06 | 0.48-2.37 | 0.881 | |  | | 43/869 | 5.0% | 0.88 | 0.55-1.42 | 0.5992 |  | 757 | 0.13 | 0.63 | 0.23-1.61 | 0.3296 |
| Standard LLIN & IRS group | 790 | 7.15 | 1.59 | 0.80-3.16 | 0.183 | |  | | 48/977 | 4.9% | 0.87 | 0.52-1.47 | 0.609 |  | 773 | 0.14 | 0.97 | 0.41-2.28 | 0.9426 |
| PBO-py LLIN & IRS group | 793 | 4.28 | 0.79 | 0.42-1.47 | 0.450 | |  | | 24/919 | 2.6% | 0.49 | 0.27-0.86 | 0.015 |  | 779 | 0.09 | 0.54 | 0.28-1.06 | 0.0724 |

*adjusted for their respective value at baseline, **one outlier removed
